# Identification of shared disease marker genes and underlying mechanisms between rheumatoid arthritis and Crohn disease through bioinformatics analysis

**DOI:** 10.1097/MD.0000000000038690

**Published:** 2024-06-28

**Authors:** Peifei Peng, Ying Shen

**Affiliations:** a Department of Geriatrics, Liyuan Hospital, Tongji Medical College, Huazhong University of Science and Technology, Wuhan, China.

**Keywords:** rheumatoid arthritis, Crohn disease

## Abstract

As chronic autoimmune inflammatory diseases, rheumatoid arthritis (RA) and Crohn disease (CD) are closely associated and display a significant positive correlation. However, the underlying mechanisms and disease markers responsible for their cooccurrence remain unknown and have not been systematically studied. Therefore, this study aimed to identify key molecules and pathways commonly involved in both RA and CD through bioinformatic analysis of public sequencing databases. Datasets for RA and CD were downloaded from the GEO database. Overlapping genes were identified using weighted gene co-expression network analysis and differential analysis crossover, and enrichment analysis was conducted for these genes. Protein-protein interaction networks were then constructed using these overlapping genes to identify hub genes. Expression validation and receiver operating characteristic curve validation were performed for these hub genes using different datasets. Additionally, the immune cell correlation, single-cell expression cluster, and the immune cell expression cluster of the core gene were analyzed. Furthermore, upstream shared microRNAs (miRNA) were predicted and a miRNA-gene network was constructed. Finally, drug candidates were analyzed and predicted. These core genes were found to be positively correlated with multiple immune cells that are infiltrated by the disease. Analysis of gene expression clusters revealed that these genes were mostly associated with inflammatory and immune responses. The miRNA-genes network analysis suggested that hsa-miR-31-5p may play an important role in the common mechanism of RA and CD. Finally, tamibarotene, retinoic acid, and benzo[a]pyrene were identified as potential treatment options for patients with both RA and CD. This bioinformatics study has identified ITGB2, LCP2, and PLEK as key diagnostic genes in patients with both RA and CD. The study has further confirmed that inflammation and immune response play a central role in the development of both RA and CD. Interestingly, the study has highlighted hsa-miR-31-5p as a potential key player in the common mechanism of both diseases, representing a new direction in research and a potential therapeutic target. These shared genes, potential mechanisms, and regulatory networks offer new opportunities for further research and may provide hope for future treatment of patients with both RA and CD.

## 1. Introduction

Rheumatoid arthritis (RA) is a persistent autoimmune inflammatory disorder impacting roughly 1% of the global populace. It may result in lasting joint impairment and functional limitations, imposing considerable societal and economic burdens.^[1–4]^ Similarly, Crohn disease (CD) is a chronic autoimmune inflammatory disorder, representing a global health concern, particularly prevalent in newly industrialized nations undergoing westernization.^[5]^ As autoimmune diseases, they share similar genetic factors, environmental triggers, and pathophysiological mechanisms.

As autoimmune diseases, they exhibit shared genetic elements, environmental triggers, and pathophysiological mechanisms.^[6,7]^ For instance, they both involve dysregulation of the immune system, including overexpression of proinflammatory cytokine tumor necrosis factor.^[8,9]^ T-cell-mediated antigen-specific pathways seem to be interconnected in both conditions. Naive T-cells undergo division and differentiation into effector T-cells, with cytokine production favoring a particular phenotype in the human leukocyte antigen and CD cell populations. Subsequently, this results in the generation of numerous cytokines and other inflammatory mediators, as well as the infiltration of immune cells such as neutrophils, macrophages, and B cells.^[10]^ Multiple epidemiological studies have shown that the risk of developing RA is more than twice as high in patients with inflammatory bowel disease compared to those without it.^[11–13]^ Similarly, subclinical intestinal inflammation has also been observed in patients with RA.^[14]^ Based on the above clinical studies, we can confirm that there is a close relationship between RA and CD. Therefore, it is necessary to study RA and CD at the genetic or biomarker level.

Advancements in sequencing technology and bioinformatics have enabled the exploration of shared disease pathogenesis at the genetic level. Weighted gene co-expression network analysis (WGCNA) is a systems biology technique aimed at discovering co-expressed gene modules, examining the correlation between gene networks and specific phenotypes, and identifying key genes within the network, all without bias.^[15,16]^ The basic principle of the WGCNA algorithm is to first calculate the correlation between all gene pairs in the dataset, and then use this information to construct a gene network. This gene network can be used to cluster genes into modules using clustering algorithms. The obtained modules can be further analyzed to identify the relationships between biological processes and to identify potential gene targets for further research.^[17]^ Therefore, it is often used to identify specific modules and potential biomarkers. This study identified the core genes associated with the cooccurrence of RA and CD and explored their correlation with immune cells. The miRNAs that regulate all core genes were predicted using an online platform, and drugs that are effective for core genes were predicted. Our research may provide a potential basis for the early diagnosis and targeted treatment of cooccurring RA and CD.

**Figure 1.**
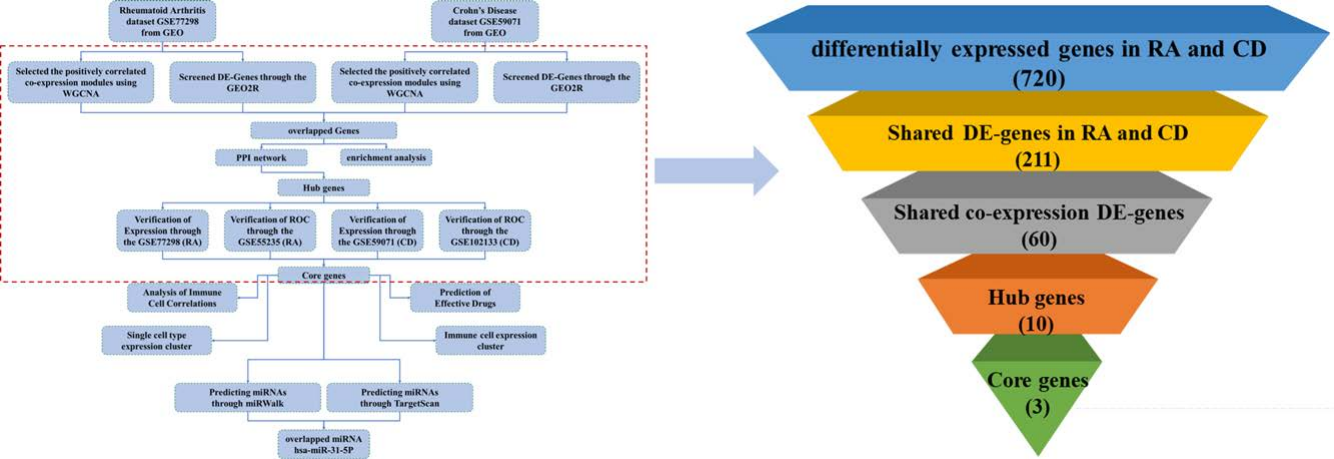
Flowchart of the design and evaluation of this study. DE = differentially expressed, GEO = gene expression omnibus, GEO2R = differential expression analysis tools, miRNA = microRNA, PPI = protein-protein interaction network, ROC = receiver operating characteristic curve, WGCNA = weighted gene co-expression network analysis.

The flowchart depicting the analysis core gene extraction curation pipeline is presented in Figure 1. Further details on each step are provided in the subsequent subsection.

## 2. Materials and methods

### 2.1. Acquisition and preprocessing of microarray data

The expression datasets GSE77298,^[18]^ GSE55235,^[19]^ GSE59071,^[20]^ and GSE102133^[21]^ for RA and CD were downloaded via NCBI GEO (http://www.ncbi.nlm.nih.gov/geo/). Among these, GSE77298 and GSE59071 were used as the training group, and GSE55235 and GSE102133 were used as the testing group. Subsequently, GEO2R was used to preprocess the datasets (https://www.ncbi.nlm.nih.gov/geo/geo2r/) to obtain differentially expressed genes (DE-Genes).^[22]^ After limiting the data by setting the parameters *P* < .05 and |logFC| ≥1, the data were visualized using Origin 2021.^[23–25]^ Venny 2.1 was then used to obtain the intersection of the DE-Genes for RA and CD (https://bioinfogp.cnb.csic.es/tools/venny/index.html).

In the above datasets, GSE77298 includes 7 healthy control individuals and 16 RA patients. GSE55235 includes 10 healthy control individuals and 10 RA patients. GSE59071 includes 11 healthy control individuals and 8 CD patients. GSE102133 includes 12 healthy control individuals and 65 CD patients (see Table 1, Supplemental Digital Content, http://links.lww.com/MD/N31, the comprehensive details of the samples utilized in this study).

### 2.2. Modular clustering analysis

In this study, the gene co-expression network was constructed by the *WGCNA* package in R4.2.1.^[17]^ In detail, the original dataset was first filtered, and the median absolute deviation values were used for sorting to select the advanced genes to remove the genetic genes that were low or unchanged. Our quality control criterion was to remove genes with a standard deviation of <0.5. Next, sample clustering and power value calculation were performed, and the optimal soft threshold was calculated after plotting a scatter plot of power values. Then, based on the topological overlap metric, the genes were clustered based on the minimum size of each gene network module set to 50 after converting the adjacency matrix into a topological overlap metric matrix. Phenotypes were calculated from the eigenvectors of each module. Modules with high correlation coefficients were considered relevant to clinical characteristics and then used for subsequent analysis.^[26]^

### 2.3. Screening and enrichment analysis of overlapping genes between RA and CD

To obtain genes associated with the occurrence of RA and CD, the upregulated genes obtained from the differential analysis of RA and CD and the positive correlation module obtained from the WGCNA analysis were de-intersected to obtain overlapping genes. These overlapping genes were further utilized for enrichment analysis. Then, we performed gene ontology (GO) annotation and Kyoto Encyclopedia of Genes and Genomes (KEGG) pathway enrichment analysis using *R* language,^[27]^ with GO analysis including cellular component, biological process, and molecular function. The protein-protein interaction (PPI) network was constructed for these overlapping genes by using the STRING (https://string-db.org/cgi/input.pl) online tool.^[28]^

### 2.4. Screening and validation of hub gene

Based on the above PPI network, center nodes were studied via the maximum cluster centrality (MCC) algorithm in CytoHubba, which was a plugin in Cytoscape.^[29]^ The first 10 positions were defined as hub genes according to the order of MCC values. Subsequently, the hub genes were validated. The obtained hub genes were validated for expression in the training and validation sets using R language, and only those hub genes that showed differential expression (*P* < .05) in both the training and validation sets were considered validated. Additionally, the reliability of these hub genes for disease diagnosis was validated for the receiver operating characteristic curve (ROC), and only those hub genes with area under curve (AUC) values >0.7 in the ROC curves of the training and validation sets were considered validated. Finally, the genes that passed the validation were identified as core genes.

### 2.5. Analysis of immune cell correlations

The single sample gene set enrichment analysis was used to obtain the immune cell scoring for each patient in the dataset,^[30]^ and the heat mapping and differential analysis of immune cells were performed. Immune cell-related line analysis was then performed by using the core genes.

### 2.6. Analysis of single-cell types and immune cell expression clusters

To further explore the relationship between core genes and immune cells and functions, the online tool Human Protein Atlas (http://www.proteinatlas.org) was used to determine the location of core genes on cells.^[31]^ Subsequently, single-cell expression clusters and immune cell expression clusters were analyzed for core genes.

### 2.7. Prediction of miRNA and construction of its interaction network with core genes

The microRNAs (miRNA) upstream of the core genes were predicted by using miRWalk 2.0 (http://mirwalk.umm.uni-heidelberg.de/) and TargetScan (https://www.targetscan.org/vert_80/),^[32,33]^ respectively. To improve the accuracy of the prediction, only genes that were predicted by both platforms could participate in the network construction. The network of the obtained miRNAs and core genes was constructed by using cytospace after taking the intersection of the miRNAs predicted by the core genes.

### 2.8. Prediction of effective drugs

To obtain effective drugs for RA and CD, the online tool Enrichr (https://maayanlab.cloud/Enrichr/) was used to predict effective drugs for core genes.^[34]^ Subsequently, the drugs predicted to be effective against the core genes simultaneously were visualized by using the online tool DrugBank (https://go.drugbank.com/).

## 3. Result

### 3.1. Screening of DE-Genes

**Figure 2.**
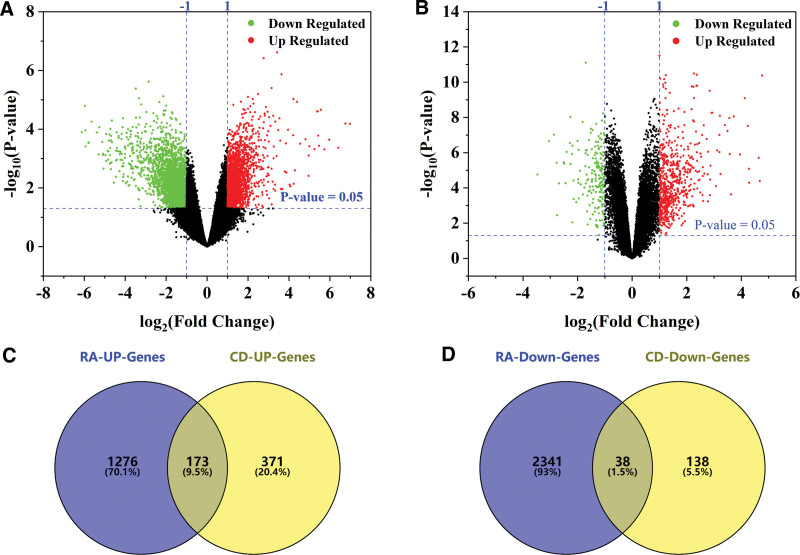
Prediction of differential genes in RA and CD. (A) Volcano plot of differentially expressed genes in RA. (B) Volcano plot of differentially expressed genes in CD. Venn diagram of (C) upregulated and (D) downregulated genes in RA and CD. CD = Crohn disease, RA = rheumatoid arthritis.

To obtain the same DE-Genes in RA and CD, differential analysis was performed on the datasets GSE77298 (RA) and GSE59071 (CD), respectively. Figure 2A is a volcano plot drawn by a dataset of RA. The cutoff criteria of the volcano plot were |logFC| ≥1.0 and *P* < .05, it was found that a total of 3788 genes were dysregulated in the dataset. Figure 2B is the volcano plot drawn for the CD patient dataset GSE59071. The cutoff criteria of the volcanic plot were the same as above, and a total of 720 genes could be found to be dysregulated in the dataset. As shown in Figure 2C, there were 173 genes upregulated in both RA and CD, while as shown in Figure 2D, 38 genes were downregulated (see Table 2, Supplemental Digital Content, http://links.lww.com/MD/N32, list of upregulated and downregulated differentially expressed genes).

### 3.2. Weighted gene co-expression network analysis

**Figure 3.**
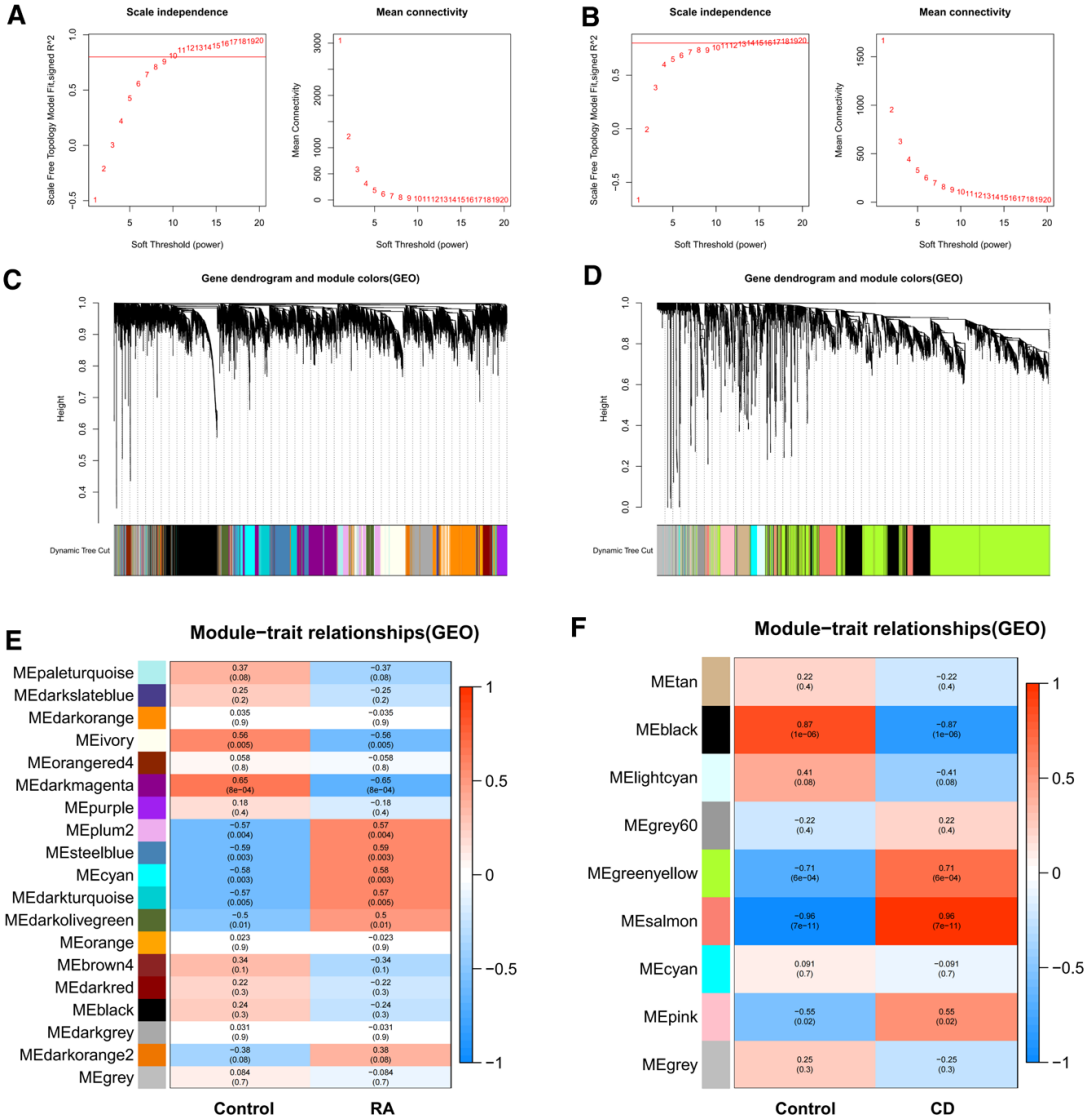
Co-expression analysis of weighted gene networks in RA and CD. (A) The determination of the soft threshold of RA, when the soft threshold is 10, *R*^2^ > 0.8, and the average connectivity is <100. (B) Determination of the soft threshold for CD, When the soft threshold is 14, *R*^2^ > 0.8, and the average connectivity is <100. (C) Cluster dendrogram of RA gene co-expression network modules. (D) Cluster dendrogram of CD gene co-expression network modules. (E) Shape relationship diagram of the modular clinical features of RA. Each row corresponds to a color and each column to a clinical trait. The number above the module represents the correlation and the value in parentheses represents the *P*-value. (F) Shape relationship diagram of the modular clinical features of CD. CD = Crohn disease, RA = rheumatoid arthritis.

To find functional clusters associated with RA and CD, the datasets GSE77298 (RA) and GSE59071 (CD) were used and a gene coexpression network was constructed by the WGCNA package. Figure 3A and B shows the scatter plots of power values for RA and CD, respectively, which were used to determine an optimal soft threshold. It can be found that in GSE77298 (RA), when the soft threshold is 10, *R*^2^ > 0.8, and average connectivity <100. In GSE59071 (CD), when the soft threshold is 14, *R*^2^ > 0.8, and average connectivity <100. It shows that the constructed network conforms to the scale-free feature subsequently, tree clusters were constructed based on the optimal soft threshold (Fig. 3C and D), and it was shown that genes in dataset GSE77298 (RA) were divided into a total of 19 modules (Fig. 3C) and genes in dataset GSE59071 (CD) were divided into a total of 9 modules (Fig. 3D). Figure 3E is the module traits of the dataset GSE77298 (RA), and it can be found that Steelblue and Cyan modules have the greatest positive correlation with RA. Figure 3F is the module traits of the dataset GSE59071 (CD), and it can be found that the Salmon and Greenyellow modules have the greatest positive correlation with CD. The above modules will also be used for subsequent analysis.

### 3.3. Screening and functional analysis of overlapping genes

**Figure 4.**
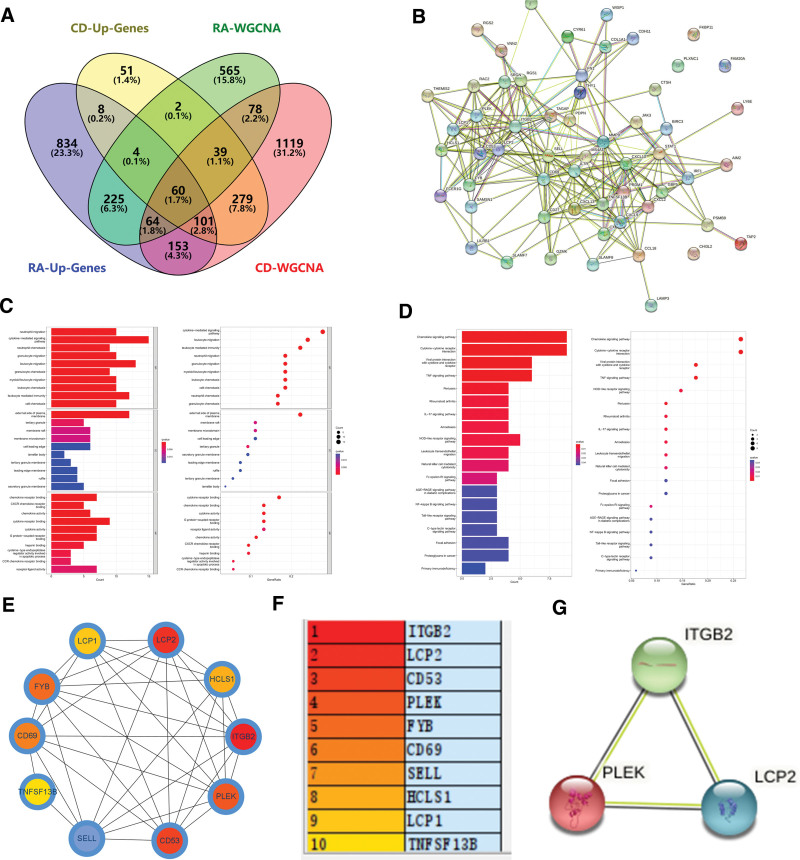
Construction and enrichment analysis of overlapping gene networks. (A) Venn diagram of upregulated differential genes and positively correlated modules in RA and CD. (B) PPI network of overlapping genes. (C) Gene ontology and (D) Kyoto Encyclopedia of Genes and Genomes enrichment analysis of overlapping genes. (E) Network diagram of hub genes. (F) Schematic diagram of hub gene. (G) PPI network diagram of core genes. CD = Crohn disease, PPI = protein-protein interaction, RA = rheumatoid arthritis.

To screen out the same related genes with RA and CD, the selected modules were crossed over with those obtained by differential analysis (Fig. 4A), resulting in 60 overlapping genes (see Table 3, Supplemental Digital Content, http://links.lww.com/MD/N33, list of 60 overlapping genes). Meanwhile, a PPI network was constructed for these 60 overlapping genes (Fig. 4B). Then these genes were subjected to enrichment analysis subsequently (Fig. 4C), which showed that in terms of cellular components, genes were mainly enriched in the external side of plasma membrane, tertiary granule, and membrane raft. In terms of biological processes, genes were mainly enriched in neutrophil migration leukocyte migration, and leukocyte-mediated immunity. In terms of molecular function, genes were mainly focused on cytokine receptor binding, cytokine activity, and chemokine receptor binding. In addition, KEGG analysis showed that the genes were mainly enriched in the Chemokine signaling pathway, tumor necrosis factor (TNF) signaling pathway, and IL-17 signaling pathway (Fig. 4D).

### 3.4. Screening and validation of hub gene

To explore the important central genes common in RA and CD, the top 10 defined as hub genes were screened according to the order of MCC values by using the plugin CytoHubba in Cytoscape (Fig. 4E). Figure 4F shows the gene symbols for hub genes. Subsequently, these hub genes were verified by using the training dataset GSE77298 (RA), the test dataset GSE55235 (RA), the training dataset GSE59071 (CD), and the test dataset GSE102133 (CD). The results showed a total of 3 genes (Fig. 4G) with positive results in the above dataset, which were finally identified as core genes. Figure S1A (see Figure S1, Supplemental Digital Content, http://links.lww.com/MD/N28, validation of gene expression) is the expression quantity map of the core gene in GSE77298 (RA), and Figure S1B (see Figure S1, Supplemental Digital Content, http://links.lww.com/MD/N28, validation of gene expression) is the expression quantity diagram of the core gene in GSE55235 (RA). A significant positive correlation between these core genes and RA could be found. Figure S1C (see Figure S1, Supplemental Digital Content, http://links.lww.com/MD/N28, validation of gene expression) showed the expression of core genes in dataset GSE59071 (CD) and Figure S1D (see Figure S1, Supplemental Digital Content, http://links.lww.com/MD/N28, validation of gene expression) showed the expression of core genes in dataset GSE102133 (CD), which could be found that these core genes had a significant positive correlation with CD. Further, the accuracy of core gene and disease diagnosis was analyzed by using the *pROC* package in R4.2.1. Figure S2A (see Figure S2, Supplemental Digital Content, http://links.lww.com/MD/N29, receiver operator curves analysis of hub genes) showed the ROC curve of the core gene in GSE77298 (RA) and Figure S2B (see Figure S2, Supplemental Digital Content, http://links.lww.com/MD/N29, receiver operator curves analysis of hub genes) showed the ROC curve of the core gene in GSE55235 (RA), which could be found that the AUC values of the core gene in rheumatoid diseases are >0.7. Figure S2C (see Figure S2, Supplemental Digital Content, http://links.lww.com/MD/N29, receiver operator curves analysis of hub genes) showed the ROC curve of the core gene in GSE59071 (CD) and Figure S2D (see Figure S2, Supplemental Digital Content, http://links.lww.com/MD/N29, receiver operator curves analysis of hub genes) showed the ROC curve of the core gene in GSE102133 (CD), and it can be found that the AUC values of the core gene in CD are likewise >0.7. In summary, the core genes are very accurate in the diagnosis of RA and CD.

### 3.5. Immune correlation analysis of core genes

**Figure 5.**
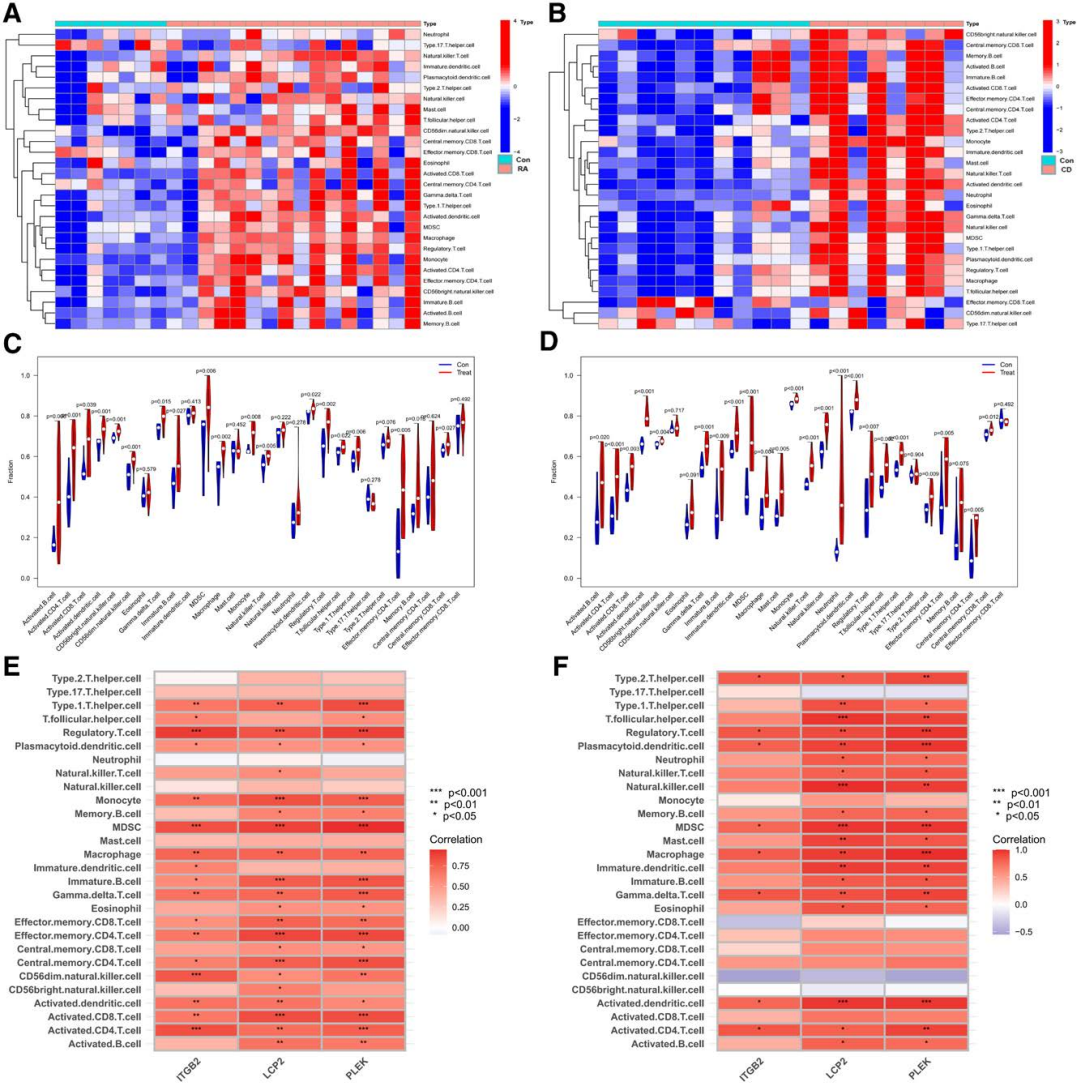
Analysis of immune cell correlation. (A) Immune cell heatmap of the RA dataset GSE77298. (B) Immune cell heatmap of the CD dataset GSE59071. (C) Immune cell differential analysis plot for the RA dataset GSE77298. (D) Immune cell differential analysis graph for CD dataset GSE59071. (E) Immune cells correlation analysis of core genes in the RA dataset GSE77298. (F) Immune cells correlation analysis of core genes in the CD inflammation dataset GSE7729. CD = Crohn disease, RA = rheumatoid arthritis.

To explore the relationship between core genes and immune cells, immune correlation analysis was performed on the datasets GSE77298 (RA) and GSE59071 (CD). Figure 5A and B is immune cell hot diagrams of datasets GSE77298 (RA) and GSE59071 (CD), respectively. Figure 5C and D is the differential analysis plots of immune cells for data sets GSE77298 (RA) and GSE59071 (CD), respectively, showed that 19 kinds of immune cells were differentially expressed in RA, and 23 kinds of immune cells were differentially expressed in CD. Figure 5E is an analysis of immune cell correlation in dataset GSE77298 (RA). Figure 5F is an analysis of immune cell correlation in the core gene in the dataset GSE59071 (CD), showing that in RA and CD, there are *Regulatory.T.cell, Plasmacytoid.dendritic.cell, MDSC, Macrophage, Gamma.delta.T.cell, Activated.CD8.T.cell, Activated.CD4.T.cell* 8 immune cells have a positive correlation with core genes. The above results suggested that most immune cells are upregulated in RA and CD, and these core genes were positively correlated with most immune cells, so it is likely that these core genes controlled the simultaneous occurrence of RA and CD by these differentially positively correlated immune cells.

### 3.6. Expression cluster analysis of core genes

**Figure 6.**
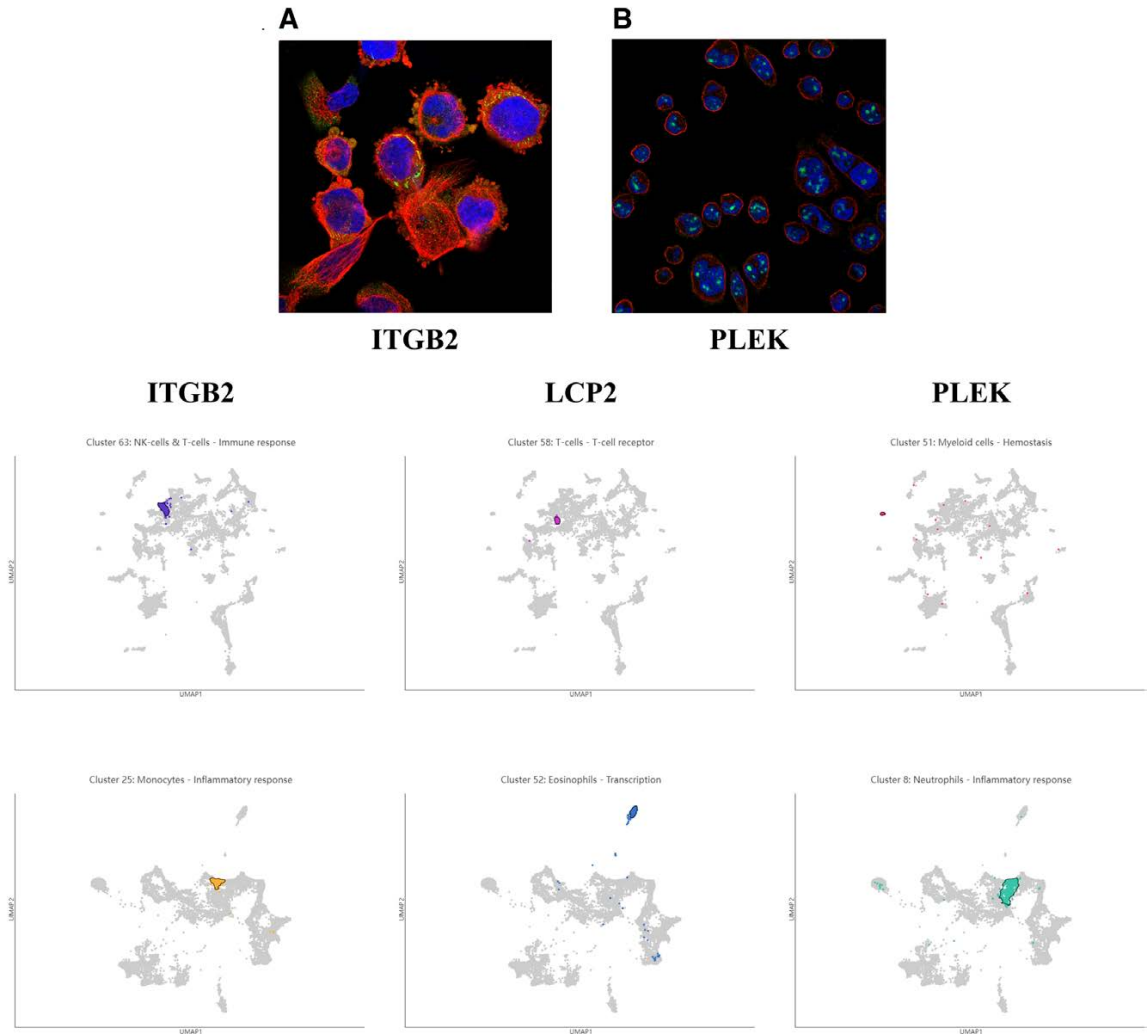
Expression cluster analysis of core genes. Cellular immunofluorescence map of (A) ITGB2 gene and (B) PLEK gene. (C) Single-cell type expression cluster analysis of core genes. (D) Immune cell expression cluster analysis of core genes.

To further understand the core genes, the location of the genes on the cell was queried via the online platform The Human Protein Atlas. Figure 6A is the cell immunofluorescence dyeing diagram of the ITGB2 gene, showing that ITGB2 was mainly localized in the plasma membrane. Figure 6B is mainly the cell immunofluorescent staining diagram of the PLEK gene, showing that PLEK was mainly localized to nucleoli. Unfortunately, no cellular localization profiles and information on LCP2 were sought within the platform. Also, these core genes were classified and functionally analyzed. Figure 6C shows the single-cell type expression cluster analysis of the core gene, and Figure 6D shows the immune cell expression cluster analysis of the core gene The results of the single-cell type expression cluster showed that the ITGB2 gene was mainly classified as part of NK-cells and T-cells and was associated with the immune response; the LCP2 gene was mainly classified as part of T-cells and was associated with the T-cell receptor, and the PLEK gene was mainly classified as part of myeloid cells and was associated with hemostasis. The results of the immune cell expression cluster showed that the ITGB2 gene was mainly classified in the part of monocytes, which is related to inflammatory response; the LCP2 gene was mainly classified in the part of eosinophils, which is related to transcription; and the PLEK gene was mainly classified in the part of neutrophils, which is related to the inflammatory response.

### 3.7. Construction of miRNA-core gene network

**Figure 7.**
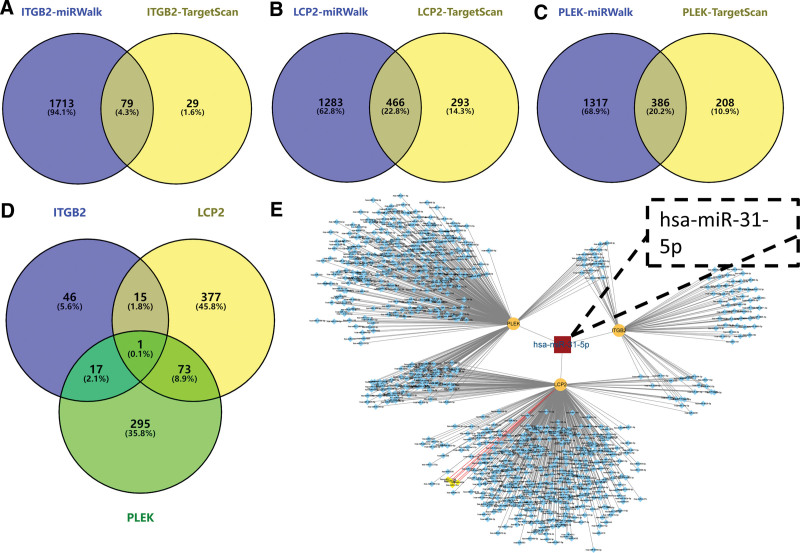
The miRNA prediction of core genes. The miRNA prediction of the (A) ITGB2 gene, (B) LCP2 gene, and (C) PLEK gene. (D) Predicted miRNA Venn diagram. (E) Network diagram of constructed miRNA-core genes. miRNA = microRNA.

To find miRNAs upstream of core genes that regulate the simultaneous occurrence of both RA and CD, miRWalk and TargetScan platforms were used to predict miRNAs upstream of core genes simultaneously. Figure 7A is the Venn diagram of ITGB2 predicted miRNAs in miRWalk and TargetScan platforms, showing that 79 miRNAs were predicted by both platforms jointly. Figure 7B is the Venn diagram of LCP2 on miRWalk and TargetScan platforms, showing that 446 MiRNA were predicted by both platforms jointly. Figure 7C is the Venn diagram of PLEK predicted miRNAs in miRWalk and TargetScan platforms, showing that 386 miRNAs were predicted by both platforms jointly. Subsequently, these predicted miRNAs were crossed (Fig. 7D) and a miRNA was finally gotten, namely has-miR-31-5p. Meanwhile, a network map of these miRNAs and core genes was established (Fig. 7E).

### 3.8. Prediction of effective drugs

To treat patients with RA and CD at the same time, the prediction of effective drugs was performed by using the obtained core genes. Figure S3A (see Figure S3, Supplemental Digital Content, http://links.lww.com/MD/N30, prediction of effective drugs for core genes) showed the drugs predicted by using the core genes in the DrugBank database. The result showed that a total of 3 drugs had simultaneous effects on the core genes. The information on these 3 drugs was visualized subsequently. Figure S3B (see Figure S3, Supplemental Digital Content, http://links.lww.com/MD/N30, prediction of effective drugs for core genes) was the information about Tamibarotene, Figure S3C (see Figure S3, Supplemental Digital Content, http://links.lww.com/MD/N30, prediction of effective drugs for core genes) was the information about retinoic acid, and Figure S3D (see Figure S3, Supplemental Digital Content, http://links.lww.com/MD/N30, prediction of effective drugs for core genes) was the information about Benzo [a] Pyrene information.

## 4. Discussion

As chronic autoimmune inflammatory diseases, there is a close relationship between RA and CD. Over time, patients with these 2 diseases are constantly being discovered.^[35,36]^ It was found that the risk of developing RA is significantly increased in patients with CD.^[12,37,38]^ Similarly, RA plays a causal role in the pathogenesis of CD.^[39]^ At present, the complex action mechanism between RA and CD is unclear, and it has not been thoroughly and systematically analyzed. This is the first study to examine the shared genes, regulatory network, and mechanisms of RA and CD through differential analysis and WGCNA to guide early detection of the disease, timely prevention, and precisely targeted therapy.

Gene expression databases from around the world can help us better understand the pathogenesis of the interaction between RA and CD, and gene expression datasets on RA and CD have been selected from these for study. Genes shared with a positive association between RA and CD were found by using WGCNA analysis and differential expression analysis. Further, a PPI network was built by using these genes and then the top 10 mmc-ranked hub genes were found from it. To further ensure the accuracy of these genes, expression validation, and diagnostic accuracy scoring were performed by using different datasets from the database. Ultimately, the key diagnostic genes ITGB2, LCP2, and PLEK associated with RA and CD were identified by the above means.

The 60 identified overlapping genes were enriched with GO for analysis. In terms of cellular component, the external side of plasma membrane and so on is at the top of the list. In terms of biological process, neutrophil migration, cytokine-mediated signaling pathway, leukocyte migration, leukocyte-mediated immunity, leukocyte chemotaxis, and neutrophil chemotaxis are the most important, and most of these biological functions are closely related to the inflammatory response. Leukocyte migration is the most important feature of inflammatory responses. Therefore, the inflammatory response may have a dominant role in the cooccurrence of RA and inflammatory CD. In terms of molecular function, genes were mainly enriched in CXCR chemokine receptor binding, CCR chemokine receptor binding, and G protein-coupled receptor binding. Chemokines and cytokines are jointly involved in the development and progression of these 2 diseases. Among them, chemokines and their receptors play an important role in the pathophysiology of many diseases by regulating the cell migration of major inflammatory and immune players.^[40]^ Among them, the CXC chemokine family (CXCL1-17) are small secreted proteins that act by signaling through the chemokine receptor (CXCR1-8), which can attract neutrophils and lymphocytes. For example, CXCR4 of the CXCR chemokine receptor family plays a central role in the accumulation of CD4 T-cells in the synovium of RA.^[41]^

A KEGG enrichment analysis was performed to identify common inflammatory and immunomodulatory pathways in RA and CD. The top-ranked KEGG pathways include the chemokine signaling pathway, cytokine-cytokine receptor interaction, TNF signaling pathway, nucleotide oligomerization domain (NOD)-like receptor signaling pathway, and IL-17 signaling pathway. The role of TNF in inflammatory bowel disease and RA is well established, and overexpression of TNF may contribute to the development and progression of CD, severe progressive arthritis, and valvular endocarditis with aortic aneurysm,^[42]^ which may be a promising therapeutic target for patients with both rheumatoid arthritis and CD. The NOD-like receptor protein family is a group of pattern recognition receptors that mediate the initial innate immune response to cellular injury and stress.^[43]^ IL-17 drives the onset of inflammation in autoimmune diseases and potentially contributes to the long-term chronic course of the disease through immunometabolic responses.^[44]^

After expression and diagnostic evaluation by the PPI network and different datasets, ITGB2, LCP2, and PLEK were identified as key diagnostic genes. The IGTB2 gene encodes an integrin beta subunit, which heterodimerizes with various alpha chains, yielding diverse integrin complexes. Integral to cell adhesion and surface-mediated signaling, integrins are pivotal cell-surface proteins. This encoded protein significantly contributes to immune responses, and mutations in this gene result in leukocyte adhesion deficiency.^[45,46]^ Suggesting that ITGB2 may play a role in leukocyte adhesion of the inflammatory response in RA and CD. The LCP2 gene encodes an adapter protein pivotal for the T-cell antigen receptor (TCR)-activated protein tyrosine kinase pathway. Interacting with growth factor receptor-bound protein 2, this protein is implicated in facilitating TCR-mediated intracellular signal transduction, thereby positively influencing T-cell development and activation, alongside mast cell and platelet functionality.^[47,48]^ Suggesting that the LCP2 gene may play an important role in TCR-mediated intracellular signaling in RA and CD immune responses. The PLEK gene is the major protein kinase C substrate of platelets.

Suggesting that the PLEK gene plays an important role in the aggregation of platelets in RA and CD. Based on immune cell correlation analysis, these core genes were positively correlated with substantial immune cell infiltration in patients with RA and CD, of which *Regulatory.T.cell, MDSC, Macrophage, Gamma.delta.T.cell, Activated.dendritic.cell*, and *Activated.CD4.T.cell* showed a high positive correlation with all core genes simultaneously in RA and CD, again suggesting that these genes are involved in multiple immune-related mechanisms in the disease process.

Further, the miRNAs upstream of these core genes were predicted by several online tools, and it was found that these 3 core genes share the same upstream miRNA, namely hsa-miR-31-5p. It was shown that hsa-miR-31-5p also has a regulatory effect on ITGB2, LCP2, and PLEK genes, which may be an important network node in the molecular mechanism of the interaction between RA and CD. This may further provide a potential aid and basis for research, prevention, early diagnosis, and treatment of RA and CD coexistence. Also, potentially effective drugs were predicted to treat RA and CD. After prediction, tamibarotene, retinoic acid, and benzo [a] pyrene show the effectiveness of the 3 core genes at the same time, which may provide potential help for the treatment of patients with RA and CD.

## 5. Conclusion

In conclusion, our work supports the idea that immune and inflammatory responses may be common causative factors, with the CXCR chemokine receptor, CCR chemokine receptor, TNF NOD-like receptor, and IL-17 likely to be the main pro-inflammatory signals and pathways. And identifies ITGB2, LCP2, PLEK, and hsa-miR-31-5P can be used as new candidates for biomarkers or potential therapeutic targets which may provide some clues to the detailed molecular mechanisms of the gut-joint axis. However, our research is limited to studies in public databases and the results need to be further validated in cellular or animal experiments, which will be a key direction for future research.

## Author contributions

**Conceptualization:** Peifei Peng.

**Data curation:** Peifei Peng.

**Formal analysis:** Peifei Peng.

**Validation:** Peifei Peng.

**Visualization:** Peifei Peng.

**Writing—original draft:** Peifei Peng.

**Methodology:** Ying Shen.

**Supervision:** Ying Shen.

**Writing—review & editing:** Ying Shen.

## Supplementary Material












