# Supplementary material for: Identification of shared disease marker genes and underlying mechanisms between rheumatoid arthritis and Crohn disease through bioinformatics analysis
Source: Medicine (Baltimore). 2024 Jun 28;103(26):e38690. doi: 10.1097/MD.0000000000038690 (PMC11466148; doi:10.1097/MD.0000000000038690)
Supplement: Supplementary file 1 [file medi-103-e38690-s001.docx]

| Dataset | PMID | Platforms | Healthy control | Rheumatoid arthritis | Crohn's disease | Both disease |
| --- | --- | --- | --- | --- | --- | --- |
| GSE77298 | 26711533 | GPL570 | 7 | 16 | 0 | 0 |
| GSE55235 | 24690414 | GPL96 | 10 | 10 | 0 | 0 |
| GSE59071 | 26313692 | GPL6244 | 11 | 0 | 8 | 0 |
| GSE102133 | 30657881 | GPL6244 | 12 | 0 | 65 | 0 |
