# Supplementary material for: Identification of shared disease marker genes and underlying mechanisms between rheumatoid arthritis and Crohn disease through bioinformatics analysis
Source: Medicine (Baltimore). 2024 Jun 28;103(26):e38690. doi: 10.1097/MD.0000000000038690 (PMC11466148; doi:10.1097/MD.0000000000038690)
Supplement: Supplementary file 2 [file medi-103-e38690-s002.docx]

| Up-Genes |  | Down-Genes |
| --- | --- | --- |
| JAK3 |  | VLDLR |
| CEMIP |  | CDC14A |
| SPP1 |  | PPARGC1A |
| CDH11 |  | GHR |
| IGK///IGKC |  | AKR1B10 |
| CXCL13 |  | ETFDH |
| TREM1 |  | PDK4 |
| SLAMF8 |  | GABRA2 |
| PLXNC1 |  | SLC16A9 |
| LILRB3 |  | ABCA8 |
| RAC2 |  | SATB2-AS1 |
| CHI3L2 |  | EDN1 |
| TIMP1 |  | CNTN3 |
| GBP5 |  | ACAT1 |
| SNX10 |  | B3GALT1 |
| FKBP11 |  | THRB |
| SLC2A3 |  | CAPN13 |
| LY6E |  | SGK2 |
| NFKBIZ |  | MIER3 |
| CXCR4 |  | ADH1C |
| RGS1 |  | BRINP3 |
| CHST11 |  | NTRK2 |
| FN1 |  | OTOP2 |
| DOCK8 |  | HSD3B2 |
| WISP1 |  | TRHDE |
| CTSH |  | PCK1 |
| PTPRC |  | CNGA1 |
| KCNJ15 |  | CNTN4 |
| IGKC |  | PON3 |
| HCLS1 |  | TM6SF2 |
| TNFAIP6 |  | RNF152 |
| BGN |  | MT1G |
| KYNU |  | SLC38A4 |
| HLA-DPB1 |  | SLC19A3 |
| SOCS3 |  | HEPACAM2 |
| THBS2 |  | G6PC |
| ITGB2 |  | RAVER2 |
| BCL2A1 |  | FABP3 |
| FYB |  |  |
| MMP9 |  |  |
| CXCL6 |  |  |
| IL1RN |  |  |
| OSMR |  |  |
| SAMSN1 |  |  |
| ARNTL2 |  |  |
| EVI2B |  |  |
| CCR1 |  |  |
| SAMD9L |  |  |
| LILRB2 |  |  |
| LILRB1 |  |  |
| CHI3L1 |  |  |
| CSF3R |  |  |
| CALU |  |  |
| ITGAX |  |  |
| C1R |  |  |
| WIPF1 |  |  |
| IL10RA |  |  |
| NCF2 |  |  |
| AIM2 |  |  |
| STAT1 |  |  |
| THEMIS2 |  |  |
| ADGRE2 |  |  |
| KDELR3 |  |  |
| AQP9 |  |  |
| CXCL10 |  |  |
| MMP2 |  |  |
| LYZ |  |  |
| IL7R |  |  |
| TRIB2 |  |  |
| CXCL8 |  |  |
| COL5A2 |  |  |
| MMP1 |  |  |
| C2 |  |  |
| CYTIP |  |  |
| IGSF6 |  |  |
| FCER1G |  |  |
| GPX8 |  |  |
| LCP2 |  |  |
| SRGN |  |  |
| SLAMF7 |  |  |
| LYN |  |  |
| PTGS2 |  |  |
| CTSK |  |  |
| FKBP10 |  |  |
| PLA2G7 |  |  |
| ELL2 |  |  |
| PCOLCE |  |  |
| CD53 |  |  |
| CFB |  |  |
| CXCL2 |  |  |
| THY1 |  |  |
| CYR61 |  |  |
| DOCK2 |  |  |
| LUM |  |  |
| TNFSF13B |  |  |
| TAGAP |  |  |
| NCKAP1L |  |  |
| DAPP1 |  |  |
| IFI16 |  |  |
| CSF2RB |  |  |
| LY96 |  |  |
| LAPTM5 |  |  |
| MMP3 |  |  |
| SERPINA1 |  |  |
| PLEK |  |  |
| IRF1 |  |  |
| TNFAIP2 |  |  |
| PSMB9 |  |  |
| CXCL1 |  |  |
| COL3A1 |  |  |
| GLIPR1 |  |  |
| TNC |  |  |
| CD44 |  |  |
| COL6A1 |  |  |
| CCL18 |  |  |
| HLA-DPA1 |  |  |
| HLA-DMA |  |  |
| NNMT |  |  |
| SELPLG |  |  |
| CRISPLD2 |  |  |
| LCP1 |  |  |
| FCGR1CP///FCGR1B///FCGR1A |  |  |
| S100A9 |  |  |
| GNA15 |  |  |
| PRDM1 |  |  |
| SELL |  |  |
| BIRC3 |  |  |
| COL1A1 |  |  |
| PDPN |  |  |
| PCSK1 |  |  |
| HLA-DMB |  |  |
| INPP5D |  |  |
| LAMP3 |  |  |
| IFI6 |  |  |
| GZMK |  |  |
| RAB31 |  |  |
| FCGR2A |  |  |
| LEF1 |  |  |
| CD86 |  |  |
| FPR1 |  |  |
| COL4A2 |  |  |
| LPCAT1 |  |  |
| APOL1 |  |  |
| MZB1 |  |  |
| CYBB |  |  |
| MMP14 |  |  |
| SERPINA3 |  |  |
| SLAMF1 |  |  |
| LOX |  |  |
| CD180 |  |  |
| TAP2 |  |  |
| XBP1 |  |  |
| FCN1 |  |  |
| CXCL9 |  |  |
| PLAU |  |  |
| CD69 |  |  |
| SRPX2 |  |  |
| CLU |  |  |
| CD27 |  |  |
| PHLDA1 |  |  |
| MNDA |  |  |
| ST8SIA4 |  |  |
| ENTPD1 |  |  |
| RGS2 |  |  |
| SLC7A5 |  |  |
| S100A8 |  |  |
| FAM20A |  |  |
| FAP |  |  |
| C3AR1 |  |  |
| LOXL2 |  |  |
| VNN2 |  |  |
| SLFN5 |  |  |
| MS4A1 |  |  |
