# Supplementary material for: Identification of shared disease marker genes and underlying mechanisms between rheumatoid arthritis and Crohn disease through bioinformatics analysis
Source: Medicine (Baltimore). 2024 Jun 28;103(26):e38690. doi: 10.1097/MD.0000000000038690 (PMC11466148; doi:10.1097/MD.0000000000038690)
Supplement: Supplementary file 3 [file medi-103-e38690-s003.docx]

| JAK3 |
| --- |
| CDH11 |
| IGK///IGKC |
| CXCL13 |
| SLAMF8 |
| PLXNC1 |
| RAC2 |
| CHI3L2 |
| GBP5 |
| FKBP11 |
| LY6E |
| RGS1 |
| FN1 |
| WISP1 |
| CTSH |
| IGKC |
| HCLS1 |
| ITGB2 |
| FYB |
| MMP9 |
| CXCL6 |
| SAMSN1 |
| LILRB1 |
| C1R |
| AIM2 |
| STAT1 |
| THEMIS2 |
| CXCL10 |
| IL7R |
| FCER1G |
| LCP2 |
| SRGN |
| SLAMF7 |
| CD53 |
| CXCL2 |
| THY1 |
| CYR61 |
| TNFSF13B |
| TAGAP |
| PLEK |
| IRF1 |
| PSMB9 |
| CCL18 |
| LCP1 |
| FCGR1CP///FCGR1B///FCGR1A |
| PRDM1 |
| SELL |
| BIRC3 |
| COL1A1 |
| PDPN |
| LAMP3 |
| GZMK |
| TAP2 |
| CXCL9 |
| CD69 |
| CD27 |
| RGS2 |
| FAM20A |
| VNN2 |
| MS4A1 |
