# Supplementary figures and images for: Identification of shared disease marker genes and underlying mechanisms between rheumatoid arthritis and Crohn disease through bioinformatics analysis
Source: Medicine (Baltimore). 2024 Jun 28;103(26):e38690. doi: 10.1097/MD.0000000000038690 (PMC11466148; doi:10.1097/MD.0000000000038690)

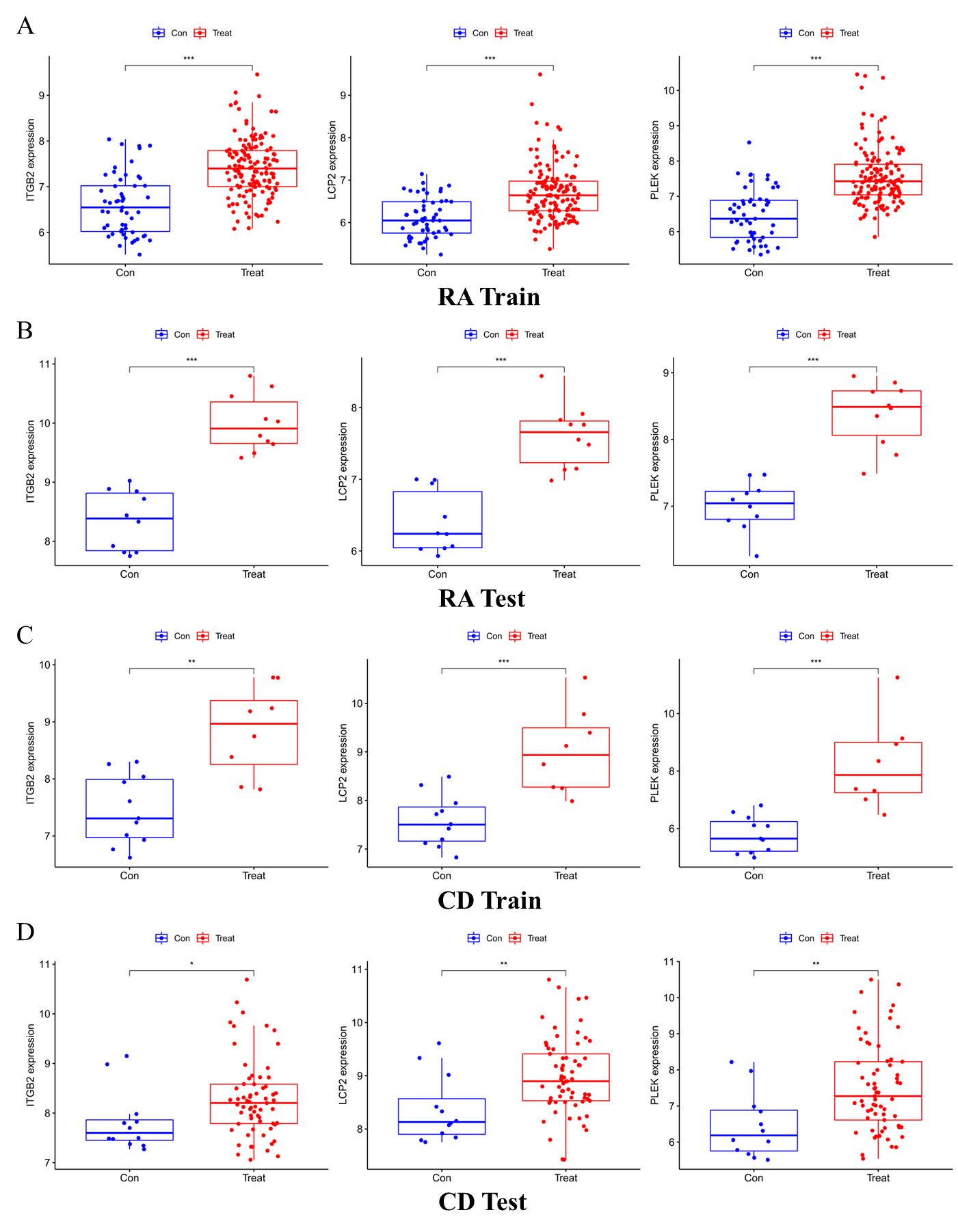

Supplement: Supplementary file 4 [file medi-103-e38690-s004.docx]

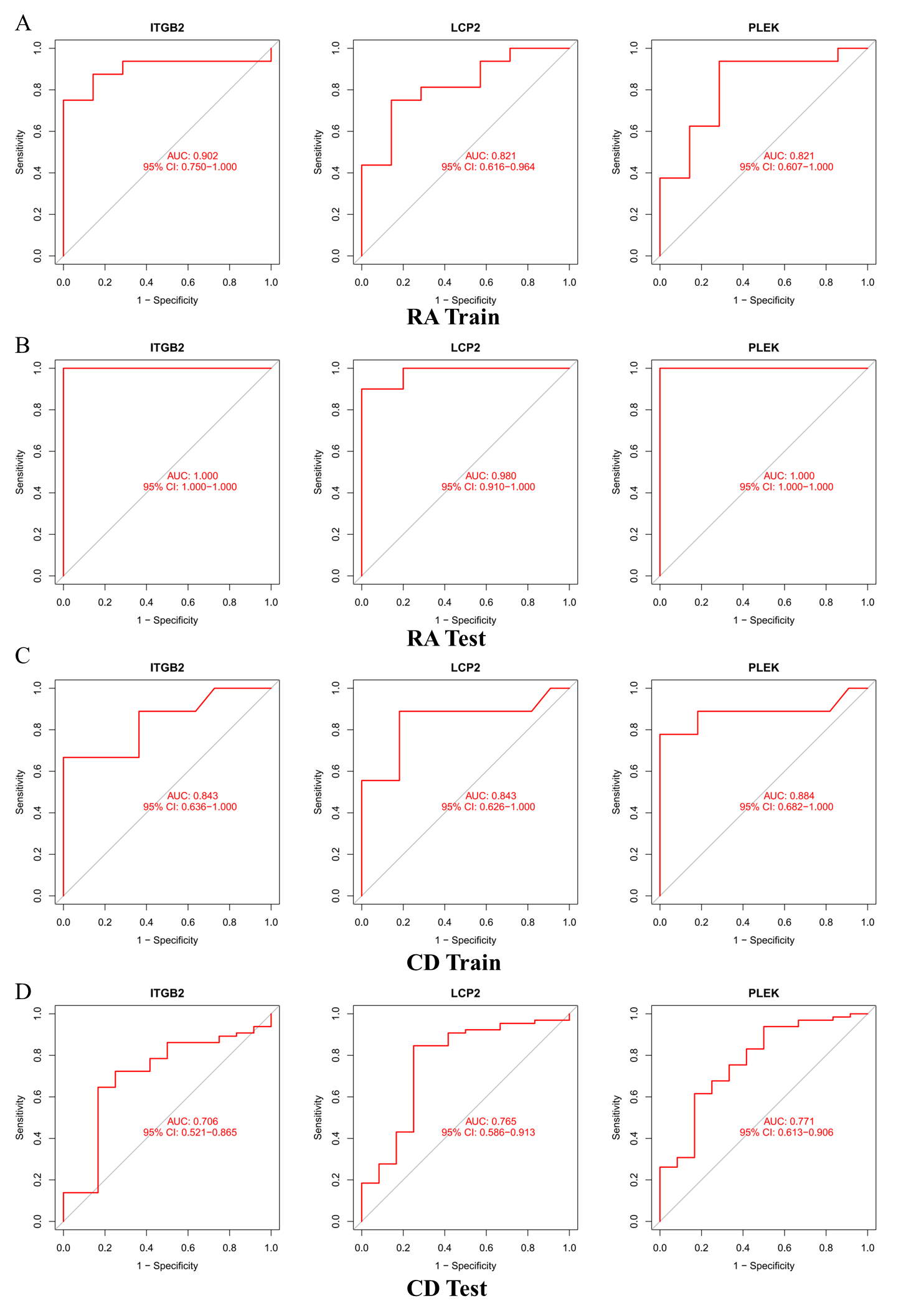

Supplement: Supplementary file 5 [file medi-103-e38690-s005.docx]

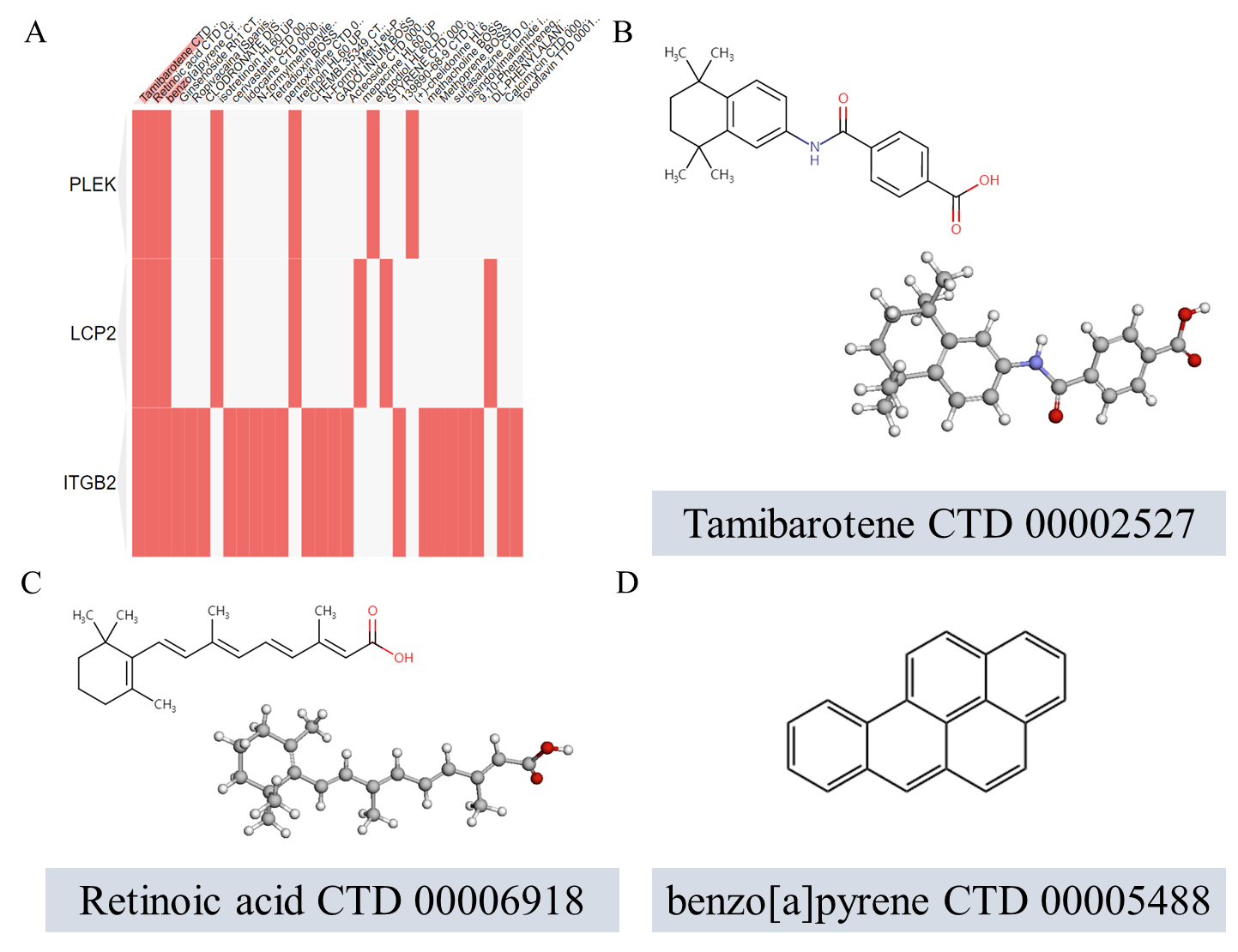

Supplement: Supplementary file 6 [file medi-103-e38690-s006.docx]
